# The feedback loop between miR-222-3p and ZEB1 harnesses metastasis in renal cell carcinoma

**DOI:** 10.1038/s41420-025-02385-0

**Published:** 2025-03-12

**Authors:** Fan Wang, Liao Li, Xiangfu Sun, Xianfu Cai, Jianjun Wang, Huiwen Luo, Yaodong Wang, Dong Ni, Decai Wang

**Affiliations:** 1 Department of Thyroid and Breast Surgery, Xiangyang No. 1 People’s Hospital, Hubei University of Medicine, Xiangyang, China; 2 Department of Child Healthcare, Xiangyang No. 1 People’s Hospital, Hubei University of Medicine, Xiangyang, China; 3 Department of Cardiothoracic Surgery, Xiangyang No. 1 People’s Hospital, Hubei University of Medicine, Xiangyang, China; 4 Department of Renal Transplantation, the First Affiliated Hospital of Xi’an Jiaotong University, Xi’an, Shaanxi Province China; 5 Department of Hepatobiliary Surgery, Mianyang Central Hospital, School of Medicine, University of Electronic Science and Technology of China, Mianyang, China; 6 NHC Key Laboratory of Nuclear Technology Medical Transformation, Mianyang Central Hospital, School of Medicine, University of Electronic Science and Technology of China, Mianyang, China; 7 Department of Urology, Mianyang Central Hospital, School of Medicine, University of Electronic Science and Technology of China, Mianyang, China; 8 Department of Urology, Union Hospital, Tongji Medical College, Huazhong University of Science and Technology, Wuhan, China

**Keywords:** Cell invasion, miRNAs

## Abstract

Renal cell carcinoma (RCC) is an aggressive malignancy originating from the renal parenchyma, often leading to high mortality due to local invasion and distant metastasis. MicroRNAs (miRNAs) play essential roles in RCC progression. Through miRNA sequencing, we identified significant upregulation of miR-222-3p in metastatic RCC tissues. Exosomes from highly metastatic RCC cells were found to transfer miR-222-3p to low-metastatic cells, enhancing their migration and invasion. Mechanistically, miR-222-3p directly targets the 3′ untranslated region (3′UTR) of the tumor-suppressor TRPS1, reducing its expression. TRPS1 downregulation releases its inhibitory effect on ZEB1, a key regulator of epithelial-mesenchymal transition (EMT), thereby promoting EMT and metastatic traits. ZEB1 further transactivates miR-222-3p, establishing a positive feedback loop. Additionally, miR-222-3p promotes a pre-metastatic niche by inducing M2 macrophage polarization, facilitating distant metastasis. These findings highlight miR-222-3p as a critical driver of RCC metastasis and suggest its potential as a diagnostic marker and therapeutic target for RCC.

## Introduction

Renal cell carcinoma (RCC) is one of the most prevalent malignancies of the genitourinary system, with 434,419 new cases and 155,702 deaths reported worldwide in 2022 [1]. Statistics indicate that approximately one-third of patients with RCC present with local or distant metastases at the time of diagnosis. Additionally, among patients undergoing nephrectomy for localized tumors, ~25% experience distant relapse [2]. Due to its high metastatic potential and resistance to conventional therapies, the prognosis for patients with metastatic RCC remains poor, with a 5-year survival rate of ~10% [3]. Understanding the molecular determinants and regulatory networks involved in RCC metastasis is crucial for developing novel diagnostic and therapeutic strategies.

MicroRNAs (miRNAs), a class of short noncoding RNAs consisting of ~22 nucleotides, are post-transcriptional regulators of gene expression that promote mRNA degradation and inhibit translation [4, 5]. Recently, several studies have confirmed that miRNAs are critical regulators of cancer initiation, proliferation, invasion, and metastasis [6–9]. Although dysregulated miRNAs have been implicated in various cellular processes during RCC pathogenesis, their specific roles and molecular mechanisms in regulating RCC metastasis remain poorly understood and require further investigation.

Exosomes are small vesicles with diameters of 40–160 nm originating from intracellular endosomes. They mediate intercellular communication between cells by transferring their cargo, including proteins, lipids, DNA, and RNAs, such as miRNA, mRNA, and tRNAs [10, 11]. Among the biomolecular cargo of exosomes, miRNAs have gained significant attention due to their high cross-species conservation and extensive regulatory effects on gene expression [12, 13]. Several studies have shown that exosome-derived miRNAs play a crucial role in tumor regulation [14, 15]. For instance, Zhang et al. demonstrated that gastric cancer-derived exosomal miR-519a-3p promotes liver metastasis by inducing M2-like macrophage polarization and angiogenesis in the liver microenvironment [16]. Hypoxic bone marrow-derived mesenchymal stem cell (BMSC)-derived exosomal miRNAs, miR-193a-5p and miR-210-3p, promote lung cancer cell metastasis by activating STAT3 and inducing epithelial-mesenchymal transition (EMT) [17]. Moreover, breast cancer cell-secreted exosomal miR-192 promotes bone metastasis by downregulating stromal CXCL12 and suppressing myeloid progenitor cell differentiation, thus enabling tumor cell colonization in the bone marrow niche [18]. In RCC, miR-222-3p has been implicated in malignancy and invasion, partly through TMP2 suppression and ERK pathway activation [19]. However, its role in facilitating intercellular communication via exosomes and its broader involvement in tumor microenvironment remodeling remain incompletely understood. Building upon existing evidence, this study identifies a TRPS1-ZEB1 positive feedback loop regulated by miR-222-3p, which drives epithelial-mesenchymal transition (EMT) and enhances metastatic traits. Furthermore, we demonstrate that exosomal miR-222-3p promotes metastasis by transferring oncogenic signals between RCC cells and inducing M2 macrophage polarization, thereby fostering pre-metastatic niche formation.

These findings reveal previously unrecognized mechanisms through which miR-222-3p orchestrates both tumor cell behavior and the metastatic microenvironment. By targeting the miR-222-3p/TRPS1/ZEB1 axis and exosome-mediated signaling pathways, our study provides a foundation for developing innovative therapeutic strategies to mitigate RCC metastasis and improve patient outcomes.

## Results

### miR-222-3p is highly expressed in metastatic RCC and is associated with a poor prognosis

**Fig. 1:**
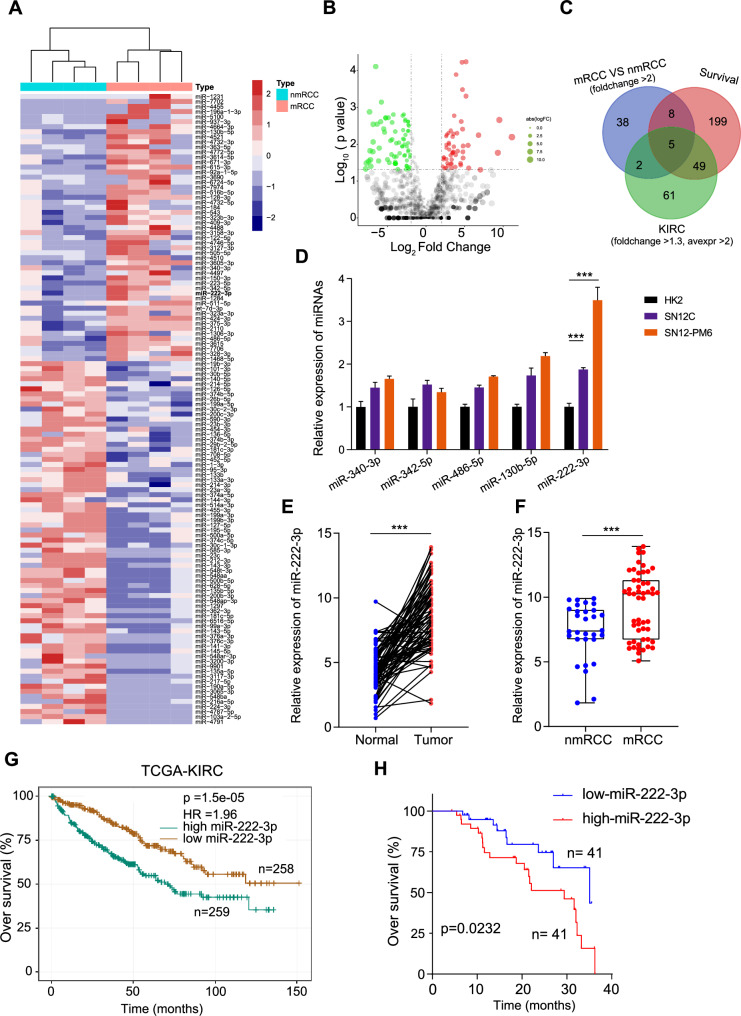
miR-222-3p is highly expressed in metastatic RCC and is associated with a poor prognosis. **A** Heatmap showing miRNAs with over two-fold differential expression in metastatic RCC compared to that in primary RCC. **B** Volcano plot illustrating the distribution of miRNAs in metastatic RCC versus primary RCC. Red dots indicate miRNAs upregulated by more than two-fold, green dots indicate miRNAs downregulated by more than two-fold, and gray dots indicate non-differential miRNAs. The vertical dashed lines represent logFC = ±1, and the horizontal dashed line represents *p* = 0.05. **C** Venn diagram depicting the overlap among three subsets. **D** Expression of five miRNAs in HK2, SN12C, and SN12-PM6 cells detected via qRT-PCR. Data are presented as the mean ± SD of three independent experiments (*n* = 3). **E** Expression of miR-222-3p in RCC tissues and adjacent normal tissues detected via qRT-PCR. Data are presented as the mean ± SD (Normal, *n* = 82; Tumor, *n* = 82). **F** Expression of miR-222-3p in metastatic versus non-metastatic RCC detected via qRT-PCR. Data are presented as the mean ± SD (nmRCC, *n* = 30; mRCC, *n* = 52) **G** Kaplan–Meier survival analysis of miR-222-3p from the TCGA dataset, stratified by miR-222-3p expression. Survival data were analyzed using the Kaplan–Meier method and log-rank test. **H** Kaplan–Meier plots of overall and disease-free survival of 82 patients with RCC, stratified by miR-222-3p expression. Survival data were analyzed using the Kaplan–Meier method and log-rank test.

To identify key miRNAs that regulate RCC metastasis, we performed miRNA sequencing on samples from four non-metastatic RCC cases and four metastatic RCC cases. Based on the sequencing results, we selected miRNAs with a variation difference of more than two-fold for heatmap analysis (Fig. 1A). The volcano plot illustrates the distribution of all miRNAs in the sequencing data (Fig. 1B). To further screen for miRNA molecules, we overlapped those upregulated by more than two-fold in our sequencing data with differentially expressed miRNAs (fold change >1.3, average expression >2) from The Cancer Genome Atlas-Kidney Renal Clear Cell Carcinoma (TCGA-KIRC) dataset, which are related to the survival prognosis of RCC, and finally obtained five miRNAs: miR-340-3p, miR-342-5p, miR-486-5p, miR-130b-5p, and miR-222-3p (Fig. 1C). Using the TCGA database, we analyzed the expression levels of these miRNAs in KIRC (Fig. S1A). Using qRT-PCR, we determined the expression of these five miRNAs in normal renal epithelial cells (HK2), low-invasive RCC cells (SN12C), and highly invasive RCC cells (SN12-PM6). The results showed that miR-222-3p was highly expressed in RCC cell lines, particularly in the highly invasive RCC cell line SN12-PM6 (Fig. 1D). Consequently, miR-222-3p was selected as the target molecule for further studies. We collected 82 RCC tissue samples and paired para-cancer tissues from the Wuhan Union Hospital and found that miR-222-3p was highly expressed in RCC (Fig. 1E). Among the 82 patients with RCC, 52 had metastatic RCC, and miR-222-3p was highly expressed in metastatic RCC (mRCC) tissues than in non-metastatic RCC (nmRCC) tissues (Fig. 1F). Moreover, survival data from TCGA-KIRC and our study indicated that high miR-222-3p expression is associated with poor survival outcomes in patients with RCC (Fig. 1G, H).

### Highly invasive RCC cells transmit invasiveness via secretion of exosomal miR-222-3p

To explore the effect of miR-222-3p in RCC cells, we generated miR-222-3p mimic and inhibitor constructs to regulate the expression of miR-222-3p and confirmed their efficiency using qRT-PCR (Fig. S2A). We used wound healing and transwell invasion assays to assess cell migration and invasion capabilities. The results showed that the migration and invasion capabilities of cells overexpressing miR-222-3p were significantly higher than those of the control group, while these effects were significantly reversed after treatment with a miR-222-3p inhibitor (Fig. S2B–E). These data revealed the role of miR-222-3p in promoting the migration and invasion of RCC cells. We found that the conditioned medium (CM) from SN12-PM6 cells significantly increased N-cadherin expression and reduced E-cadherin expression in SN12 C cells. These effects were absent in the CM from SN12-PM6 cells pretreated with an miR-222-3p inhibitor (Fig. S2F). Additionally, the CM from highly invasive SN12-PM6 cells co-cultured with SN12C cells significantly increased migration and invasion capabilities. However, this effect was not observed with the CM from SN12-PM6 cells pretreated with an miR-222-3p inhibitor (Fig. S2G–I). These findings indicate that miR-222-3p promotes RCC cell migration and invasion, and this ability can be transferred to other cells through the supernatant.

**Fig. 2:**
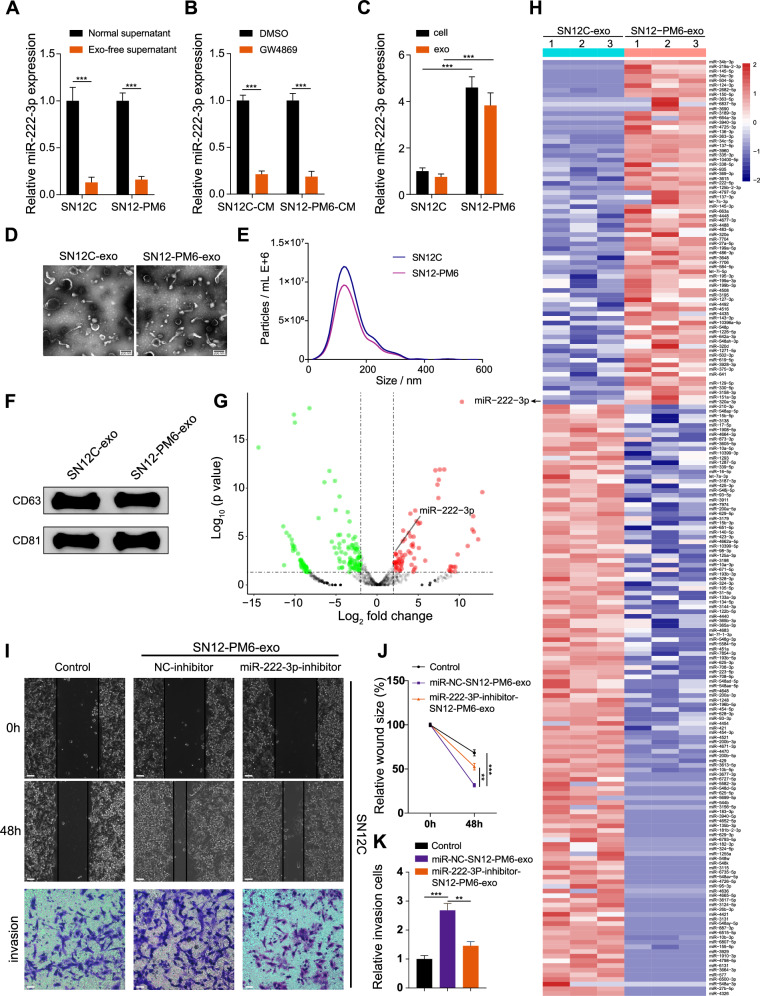
Highly invasive RCC cells transmit invasiveness via secretion of exosomal miR-222-3p. **A** Expression of miR-222-3p in normal and exosome-depleted supernatant from SN12C and SN12-PM6 detected via qRT-PCR. Data are presented as the mean ± SD of three independent experiments (*n* = 3). **B** Expression of miR-222-3p in the CM from SN12C and SN12-PM6 treated with GW4869 or DMSO. Data are presented as the mean ± SD of three independent experiments (*n* = 3). **C** Expression of miR-222-3p in cells and exosomes from SN12C and SN12-PM6 detected via qRT-PCR. Data are presented as the mean ± SD of three independent experiments (*n* = 3). **D** Electron microscopy images of exosomes from SN12C and SN12-PM6. **E** NTA of the diameter of exosomes from SN12C and SN12-PM6. **F** Western blot analysis of exosome marker proteins from SN12C and SN12-PM6. **G** Volcano plot illustrating the distribution of miRNAs in SN12-PM6-exo versus SN12C-exo. Red dots indicate miRNAs upregulated by more than two-fold, green dots indicate miRNAs downregulated by more than two-fold, and gray dots indicate non-differential miRNAs. The vertical dashed lines represent logFC = ±1, and the horizontal dashed line represents *p* = 0.05. **H** Heatmap showing miRNAs with over two-fold differential expression in SN12-PM6-exo compared to SN12C-exo. **I**–**K** Migration and invasion of SN12-PM6-exo co-cultured with SN12C cells pre-transfected with an miR-222-3p or NC inhibitor (**I**). Scale bar: 200 μm. Quantification of cell invasion (**J**). Quantification of cell migration (**K**). Data are presented as the mean ± SD of three independent experiments (*n* = 3).

To further investigate miR-222-3p presence in the supernatant, we measured its expression in both the normal supernatant and exosome-depleted supernatant. miR-222-3p was primarily expressed in exosomes of RCC cells (Fig. 2A). We treated RCC cells with the exosome inhibitor GW4869, which inhibits the release of exosomes [20], and found that the expression of miR-222-3p was significantly reduced in the CM of exosome-depleted RCC cells (Fig. 2B). Notably, miR-222-3p levels in the CM of RCC cells remained unchanged following RNase A treatment but significantly decreased with RNase A combined with Triton X-100 treatment (Fig. S3A). We detected the expression of miR-222-3p in RCC cells and their secreted exosomes and found that miR-222-3p mainly exists in exosomes (Fig. 2C). Following treatment of SN12C and SN12-PM6 cells with an miR-222-3p inhibitor, the levels of miR-222-3p in the secreted exosomes were significantly diminished (Fig. S3B, C). We identified exosomes derived from RCC cells using electron microscopy (Fig. 2D) and NTA (Fig. 2E) and detected the expression of exosomal protein markers CD63 and CD81 via western blotting (Fig. 2F). Subsequently, we performed miRNA sequencing analysis on SN12C-exo and SN12-PM6-exo. Heat maps and volcano plots showed miRNAs with more than four-fold differential expression, among which miR-222-3p was upregulated in SN12-PM6-exo (Fig. 2G, H). Next, we verified whether miR-222-3p could transfer invasiveness through exosomes. SN12-PM26-exo incubated with SN12C did not affect the expression of pre-miR-222-3p (Fig. S3D), whereas SN12-PM6-exo incubated with SN12C showed significantly enhanced migration and invasion abilities, but this effect was weakened by treatment with an miR-222-3p inhibitor (Fig. 2I–K). Thus, highly invasive SN12-PM6 cells can transfer invasiveness to less invasive SN12C cells by secreting exosomes containing miR-222-3p.

### miR-222-3p exerts oncogenic effects by inhibiting TRPS1 expression

**Fig. 3:**
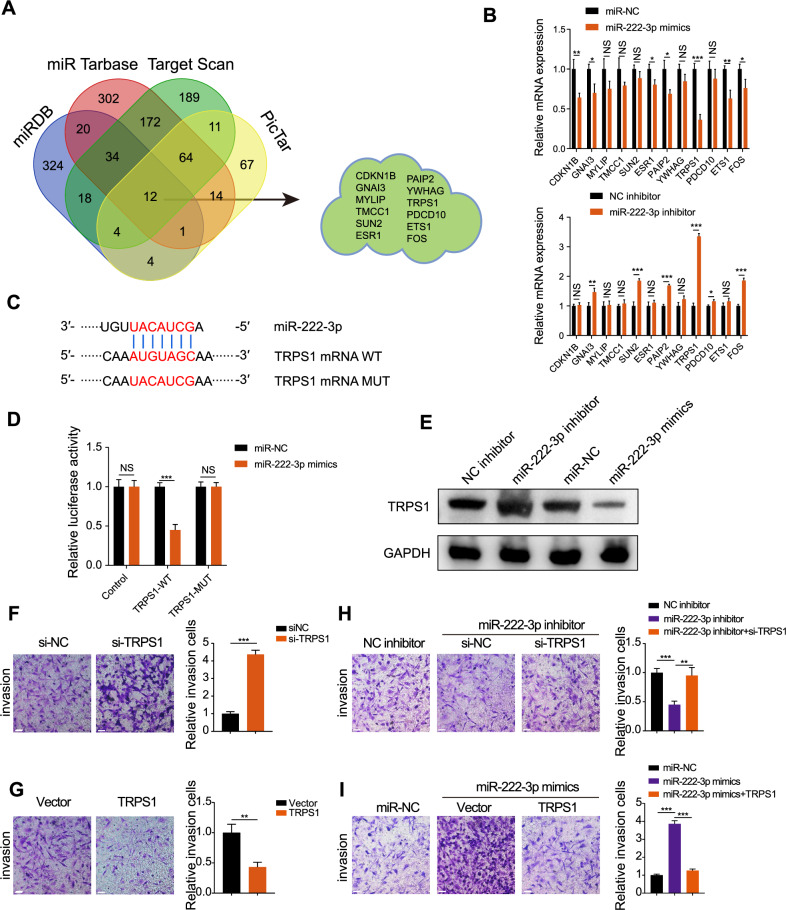
miR-222-3p exerts oncogenic effects by inhibiting TRPS1 expression. **A** Venn diagram showing the intersection of four miRNA downstream target databases. **B** Changes in mRNA expression of 12 potential downstream targets after overexpression or inhibition of miR-222-3p. Data are presented as the mean ± SD of three independent experiments (*n* = 3). **C** Schematic diagram of the binding site and mutation of miR-222-3p and TRPS1. **D** Effect of miR-222-3p transfection on the luciferase activity of TRPS1-WT or TRPS1-MUT constructs. Data are presented as the mean ± SD of three independent experiments (*n* = 3). **E** Effect of miR-222-3p inhibitor or mimics and corresponding negative controls on TRPS1 protein levels. Effect of TRPS1 knockdown (**F**) or overexpression (**G**) on the invasion of RCC SN12C cells (left). Quantification of invasive cells (right). Data are presented as the mean ± SD of three independent experiments (*n* = 3). Scale bar: 200 μm. **H** Effect of co-transfection of miR-222-3p inhibitor with si-NC or siTRPS1 on the invasion of RCC SN12C cells (left). Quantification of invasive cells (right). Data are presented as the mean ± SD of three independent experiments (*n* = 3). Scale bar: 200 μm. **I** Effect of co-transfection of miR-222-3p mimics with vector or TRPS1 on the invasion of RCC SN12C cells (left). Quantification of invasive cells (right). Data are presented as the mean ± SD of three independent experiments (*n* = 3). Scale bar: 200 μm.

To identify downstream targets of miR-222-3p, we analyzed data from four miRNA target databases: miRDB, miRTarbase, TargetScan, and PicTar. A Venn diagram identified 12 potential downstream targets (Fig. 3A). We detected their expression in RCC cells using qRT-PCR. Overexpression of miR-222-3p decreased the expression of most targets, with TRPS1 showing the most significant downregulation (Fig. 3B). Conversely, knocking down miR-222-3p using an miR-222-3p inhibitor led to the upregulation of GNAI3, SUN2, PAIP2, TRPS1, and FOS, with TRPS1 showing the most significant upregulation (Fig. 3C). Therefore, we selected TRPS1 as the downstream target of miR-222-3p. To confirm this, we used luciferase vectors containing wild-type or mutated binding sites. As shown in Fig. 3D, we transfected miR-222-3p mimics or miR-NC into SN12C cells and found that the luciferase activity of the 3′ untranslated region of TRPS1 was markedly reduced in the group co-transfected with miR-222-3p mimics and TRPS1 wild-type binding site vectors, whereas no significant changes were observed in the control or TRPS1 mutant binding site groups. Moreover, miR-222-3p knockdown significantly increased TRPS1 protein levels, whereas miR-222-3p overexpression significantly decreased them (Fig. 3E). Bioinformatic analysis indicated that TRPS1 expression was low in RCC (Fig. S4A). Moreover, survival analysis showed that low TRPS1 expression in patients with RCC was associated with poor overall and disease-free survival (Fig. S4B). Pan-cancer analysis revealed a low expression of miR-222-3p in three different types of kidney cancer: kidney chromophobe carcinoma, KIRC, and kidney renal papillary cell carcinoma (Fig. S4C). TRPS1 knockdown significantly enhanced RCC cell invasiveness (Fig. 3F) and reversed the reduction in invasiveness caused by miR-222-3p inhibition (Fig. 3H). Conversely, TRPS1 overexpression reduced invasiveness and reversed the enhanced invasiveness of RCC cells caused by miR-222-3p overexpression (Fig. 3G–I). These findings suggest that miR-222-3p regulates the invasiveness of RCC cells by targeting TRPS1.

### TRPS1 inhibits EMT in RCC cells by transcriptionally repressing ZEB1

**Fig. 4:**
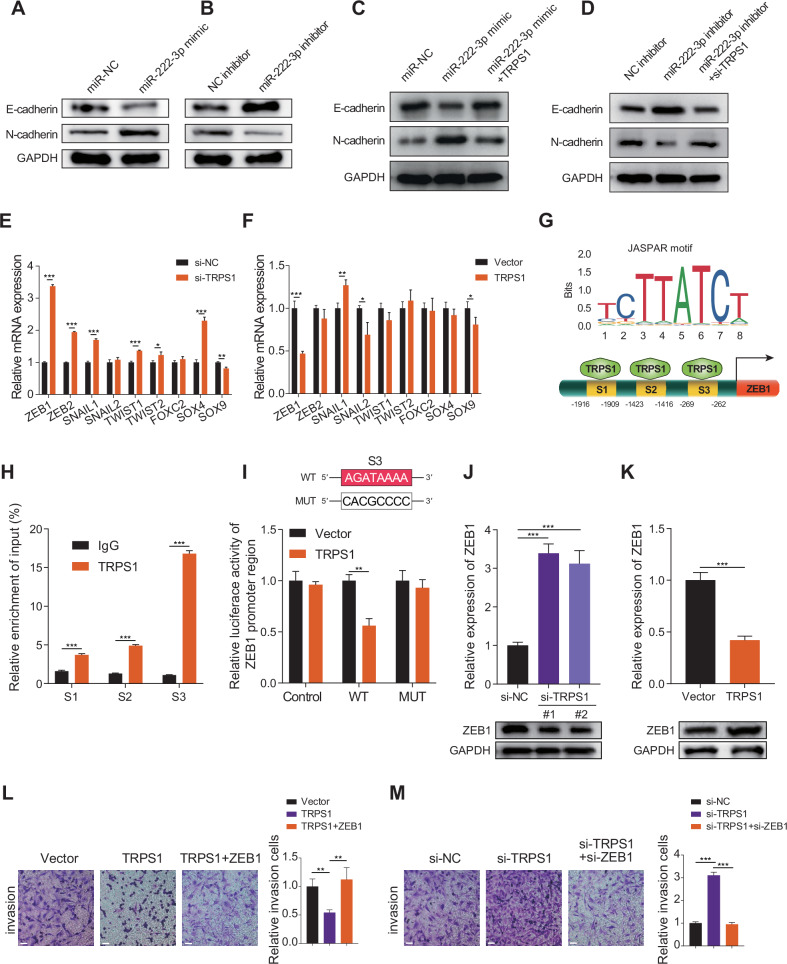
TRPS1 inhibits EMT in RCC cells by transcriptionally repressing ZEB1. **A**,**B** Migration and invasion of RCC cells transfected with miR-222-3p mimics or inhibitor and corresponding negative controls. Data are presented as the mean ± SD of three independent experiments (*n* = 3). **C**,**D** Effect of transfection with miR-222-3p mimics alone or co-transfection with TRPS1 on E-cadherin and N-cadherin expression (**C**). Effect of transfection with miR-222-3p inhibitor alone or co-transfection with si-TRPS1 on E-cadherin and N-cadherin expression (**D**). Data are presented as the mean ± SD of three independent experiments (*n* = 3). **E**,**F** mRNA expression of EMT-TFs after TRPS1 knockdown or overexpression. Data are presented as the mean ± SD of three independent experiments (*n* = 3). **G** Predicted binding motifs of TRPS1 (upper) and schematic diagram of TRPS1 binding to the ZEB1 promoter region (lower) based on the JASPAR database. **H** ChIP assay detecting TRPS1 binding to three sites on the ZEB1 promoter. Data are presented as the mean ± SD of three independent experiments (*n* = 3). **I** Schematic diagram of the S3 binding site mutation (upper). Dual luciferase reporter assay showing the effect of TRPS1 or vector transfection on luciferase activity of the ZEB1 promoter region (lower). Data are presented as the mean ± SD of three independent experiments (*n* = 3). Effect of TRPS1 knockdown (**J**) or overexpression (**K**) on ZEB1 mRNA and protein levels. Data are presented as the mean ± SD of three independent experiments (*n* = 3). **L** Effect of transfection with vector, TRPS1, or co-transfection with ZEB1 on the invasion of RCC SN12C cells (left). Quantification of invasive cells (right). Data are presented as the mean ± SD of three independent experiments (*n* = 3). Scale bar: 200 μm. **M** Effect of transfection with si-NC, si-TRPS1, or co-transfection with si-ZEB1 on the invasion of RCC SN12C cells (left). Quantification of invasive cells (right). Data are presented as the mean ± SD of three independent experiments (*n* = 3). Scale bar: 200 μm.

EMT is a biological process where epithelial cells transform into mesenchymal cells, playing a crucial role in tumor cell metastasis and invasion [21, 22]. Overexpression of miR-222-3p reduced E-cadherin expression and increased N-cadherin expression (Fig. 4A), whereas miR-222-3p inhibition had the opposite effect (Fig. 4B). This suggests that miR-222-3p promotes EMT in RCC cells. Furthermore, TRPS1 overexpression weakened EMT induced by miR-222-3p overexpression (Fig. 4C). Conversely, TRPS1 knockdown counteracted the weakening of EMT caused by miR-222-3p inhibitors (Fig. 4D). EMT is regulated by a set of EMT transcription factors (EMT-TFs) [23, 24]. We examined the effect of TRPS1 on EMT-TFs, and the results showed that after TRPS1 knockdown, the expression of ZEB1, ZEB2, SNAIL1, and SOX4 was significantly upregulated (Fig. 4E), and after TRPS1 overexpression, the expression of ZEB1 and SNAIL2 was significantly downregulated (Fig. 4F). Therefore, we selected ZEB1, which showed the most significant changes, as a potential downstream target of TRPS1. Bioinformatics analysis revealed that ZEB1 expression was elevated in RCC and was associated with poor overall and disease-free survival in patients with RCC (Fig. S5A, B). Interestingly, pan-cancer analysis showed that ZEB1 was upregulated only in KIRC but expressed at low levels in the other two types of RCC (Fig. S5C). TRPS1, a member of the GATA transcription factor family, is known to be a transcriptional repressor [25]. We investigated whether TRPS1 affects the expression of ZEB1 through transcriptional regulation. By browsing the UCSC Genome Browser website, we found that TRPS1 may bind to the promoter region of ZEB1 (Fig. S5D). Using the JASPAR tool [26], we identified the binding motif of TRPS1 and three binding sites (site1–site3) of TRPS1 in the promoter region of ZEB1 (Fig. 4G). To verify the binding of TRPS1 to the promoter region of ZEB1, we performed ChIP experiments and found that TRPS1 was mainly enriched at the S3 site in the promoter region of ZEB1 (Fig. 4H). Next, we constructed a dual-luciferase reporter gene vector containing the wild-type and S3 mutant promoter regions of ZEB1 and transfected them into RCC cells. The results showed that after TRPS1 overexpression, the promoter luciferase activity of ZEB1 significantly decreased, while no significant effect was observed on the luciferase activity in the S3 mutant vector (Fig. 4I). This suggests that TRPS1 primarily inhibits the transcriptional activity of ZEB1 by binding to the S3 site of the promoter region of ZEB1. Moreover, TRPS1 knockdown significantly increased the mRNA and protein levels of ZEB1 (Fig. 4J), whereas TRPS1 overexpression had the opposite effect (Fig. 4K). TRPS1 affects the invasiveness of RCC cells by regulating the expression of ZEB1. Overexpression of ZEB1 reversed the reduction in the invasiveness of RCC cells caused by TRPS1 overexpression (Fig. 4L), whereas ZEB1 knockdown weakened the enhanced invasiveness of RCC cells caused by TRPS1 knockdown (Fig. 4M). These findings suggest that TRPS1 inhibits EMT in RCC cells, thereby reducing their invasiveness by transcriptionally repressing ZEB1.

### miR-222-3p promotes macrophage M2 polarization and pre-metastatic niche formation

**Fig. 5:**
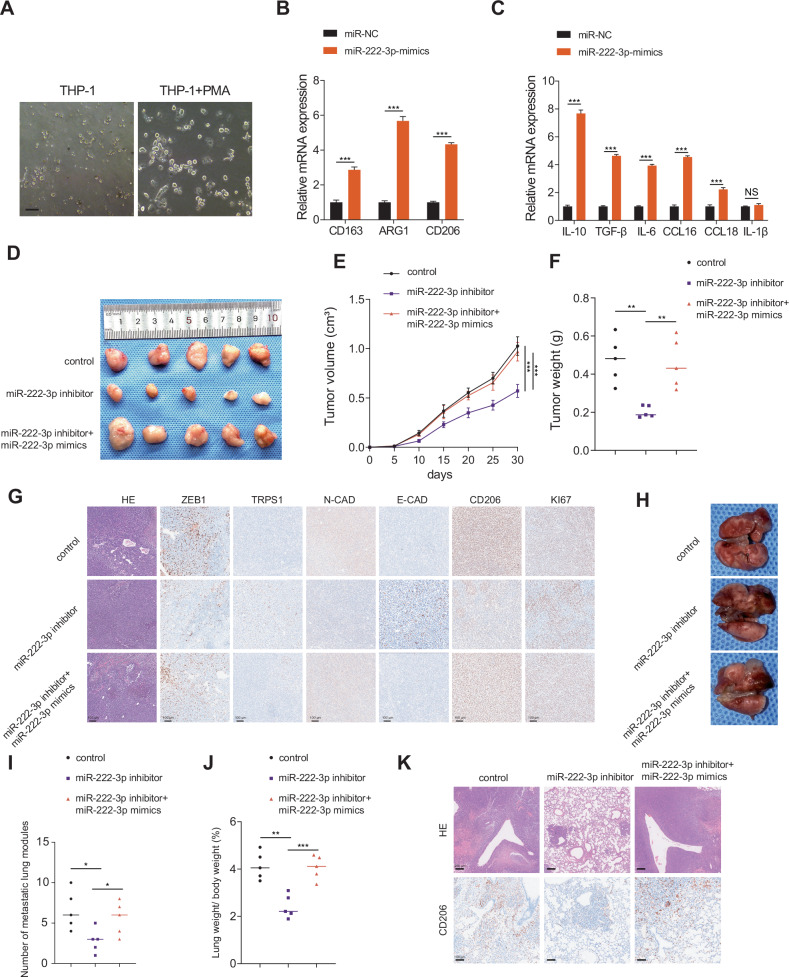
miR-222-3p promotes macrophage M2 polarization pre-metastatic niche formation. **A** Morphological images of THP-1 and THP-1 induced with PMA for 24 h, as observed via microscopy. Scale bar: 100 μm. **B** Expression of M2 markers CD163, ARG1, and CD206 in macrophages transfected with miR-222-3p mimics. Data are presented as the mean ± SD of three independent experiments (*n* = 3). **C** Changes in mRNA levels of cytokines secreted by macrophages after transfection with miR-222-3p mimics. Data are presented as the mean ± SD of three independent experiments (*n* = 3). **D**–**F** Morphological characteristics, tumor weight, and tumor volume of subcutaneous tumors in the indicated groups. Data are presented as the mean ± SD of three independent experiments (*n* = 5). **G** IHC analysis of E-cadherin, N-cadherin, CD206, TRPS1, ZEB1, and Ki67 protein expression in tumors from the indicated groups. Scale bar: 100 μm. **H** Representative images of lung metastasis in Balb/c mice from the indicated groups. Scale bar: 200 μm. **I** Number of metastatic nodules in each indicated group. Data are presented as the mean ± SD of three independent experiments (*n* = 5). **J** Lung weight/body weight (%) in each indicated group. Data are presented as the mean ± SD of three independent experiments (*n* = 5). **K** Analysis of lung metastasis by HE and IHC for CD206 in mice.

Macrophage polarization plays a significant role in tumor progression. Specifically, M1 macrophages have a protective function, whereas M2 macrophages facilitate tumor progression and metastasis [27, 28]. Existing research indicates that miR-222-3p, found in exosomes secreted by endothelial cells in breast cancer, promotes the polarization of macrophages toward the M2 phenotype [29]. However, the role of miR-222-3p in RCC remains unclear. Therefore, we hypothesized that miR-222-3p promotes M2 macrophage polarization in RCC. To test this, THP-1 cells were induced with phorbol 12-myristate 13-acetate (PMA) for 24 h to obtain macrophages (Fig. 5A), followed by transfection with miR-NC or miR-222-3p mimics. As shown in Fig. 5B, overexpression of miR-222-3p led to a significant increase in the M2 macrophage markers, such as CD206, CD163, and ARG1. Additionally, the secretion of cytokines, including IL-10, TGF-β, and IL-6 by M2 macrophages significantly increased following miR-222-3p overexpression (Fig. 5C). To verify the in vitro effects of miR-222-3p, we conducted in vivo experiments using mice. First, we compared the sequences of miR-222-3p in mice and humans and found them to be highly conserved (Fig. S6A). Using the TargetScan database, we confirmed that the target sites of miR-222-3p are conserved across different species (Fig. S6B). We then established subcutaneously transplanted tumors using Renca mouse RCC cells. The results showed that tumor volume and weight significantly decreased in the group treated with an miR-222-3p inhibitor, whereas co-transfection with miR-222-3p mimics reversed the tumor-suppressive effects of the miR-222-3p inhibitor (Fig. 5D–F). Furthermore, subcutaneous tumors were excised, and histological staining was performed. The results showed that, compared to those in the control group, the expression of N-cadherin, Ki67, CD206, and ZEB1 was significantly decreased in the miR-222-3p inhibitor treatment group, whereas the expression of E-cadherin and TRPS1 was increased (Fig. 5G). Co-transfection with miR-222-3p inhibitor and miR-222-3p mimics reversed these effects (Fig. 5G). These findings suggest that miR-222-3p promotes tumor proliferation and M2 macrophage infiltration in vivo, thereby creating a microenvironment that facilitates metastasis. To further investigate the role of miR-222-3p in tumor metastasis, we established a metastasis model by injecting mice via the tail vein. The mice were injected with 4 × 10^6^ Renca cells transfected with the miR-222-3p inhibitor, control, or co-transfected with miR-222-3p mimic. Compared to the control group, the number of lung metastases and the lung weight/body weight ratio significantly decreased after miR-222-3p inhibitor treatment; however, these effects were reversed by co-transfection with miR-222-3p mimics (Fig. 5H–J). Additionally, histological staining of the lung tissue showed that the infiltration of CD206^+^ M2 macrophages was the highest in the control group and significantly reduced after miR-222-3p inhibitor treatment (Fig. 5K). In summary, miR-222-3p promotes tumor growth in vivo and facilitates macrophage polarization toward the M2 phenotype, thereby promoting tumor metastasis and progression.

### miR-222-3p is regulated by ZEB1 in RCC

**Fig. 6:**
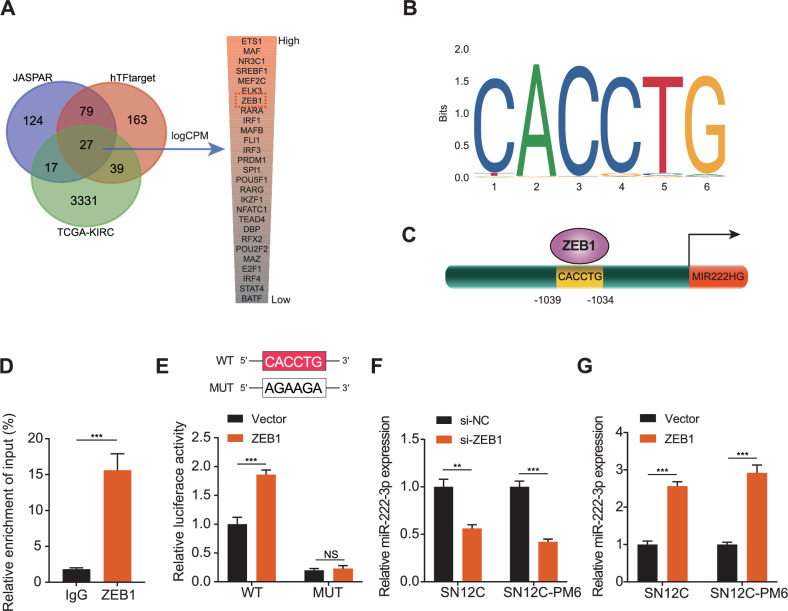
miR-222-3p is regulated by ZEB1 in RCC. **A** Venn diagram showing the intersection of transcription factors predicted by the JASPAR and hTFtarget databases with differentially expressed genes in TCGA-KIRC. **B** Binding motifs of ZEB1 obtained from the JASPAR database. **C** Schematic diagram of ZEB1 binding to the promoter region of miR-222-3p’s host gene. **D** ChIP assay detecting ZEB1 binding to the promoter region of miR-222-3p’s host gene. Data are presented as the mean ± SD of three independent experiments (*n* = 3). **E** Schematic diagram of the binding site mutation (upper). Dual luciferase reporter assay showing the effect of ZEB1 or vector transfection on luciferase activity of the promoter region of miR-222-3p’s host gene (lower). Data are presented as the mean ± SD of three independent experiments (*n* = 3). **F**, **G** Effect of ZEB1 knockdown or overexpression on miR-222-3p expression in SN12C and SN12-PM6 cells. Data are presented as the mean ± SD of three independent experiments (*n* = 3).

Next, we aimed to elucidate the potential mechanisms underlying miR-222-3p upregulation in RCC. By intersecting the JASPAR and hTFtarget databases with the differentially expressed genes in TCGA-KIRC, we identified 27 transcription factors upregulated in KIRC (Fig. 6A). Ranked in descending order of logarithm of counts per million read (logCPM) values, we unexpectedly found that ZEB1 was a potential transcription factor for miR-222-3p. Using the JASPAR database, we located a motif for ZEB1 and discovered potential ZEB1 binding regions in the promoter region of the miR-222-3p parent gene (Fig. 6B, C). Subsequently, ChIP assays confirmed that ZEB1 antibodies significantly enriched the promoter region of miR-222-3p (Fig. 6D). Furthermore, dual-luciferase reporter gene assays revealed that ZEB1 overexpression significantly enhanced miR-222-3p promoter activity, whereas mutations in the ZEB1 binding region abolished this effect (Fig. 6E). ZEB1 knockdown reduced miR-222-3p expression, whereas ZEB1 overexpression elevated it (Fig. 6F, G). These results demonstrate that miR-222-3p and ZEB1 form a mutually regulatory positive feedback loop in RCC.

### miR-222-3p is highly expressed in the blood of patients with RCC

**Fig. 7:**
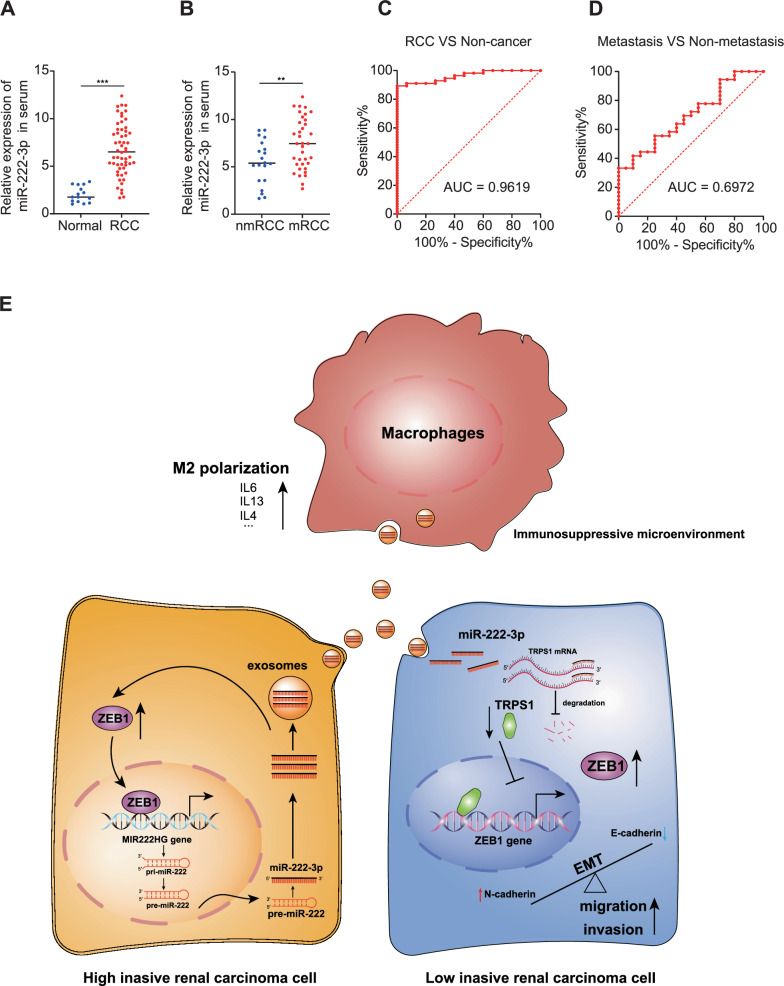
miR-222-3p is highly expressed in the blood of patients with RCC. **A** Expression of miR-222-3p in RCC tissues and adjacent normal tissues detected via qRT-PCR (Normal, *n* = 15; RCC, *n* = 56). **B** Expression of miR-222-3p in metastatic and primary RCC tissues detected via qRT-PCR (Normal, *n* = 20; RCC, *n* = 36). Receiver operating characteristic (ROC) analysis of miR-222-3p expression showing discrimination between non-tumor and tumor tissues (**C**) and between metastasis and non-metastasis (**D**). The area under the curve (AUC) is plotted as sensitivity (%) vs. 100% specificity. **E** Illustration depicting how highly invasive RCC cells promote EMT, migration, and invasion of low-invasive RCC cells via exosomal miR-222-3p. It further elucidates the enhancement of M2 macrophage polarization, which fosters the development of a pre-metastatic niche, thereby facilitating tumor metastasis and colonization.

To examine miR-222-3p expression in human RCC tissues, we used qRT-PCR to analyze blood samples collected from 82 patients with RCC and healthy controls. miR-222-3p was highly expressed in the blood of patients with RCC; notably, the level of miR-222-3p was significantly higher in patients with metastatic RCC than in those with non-metastatic RCC (Fig. 7A, B). Subsequently, we plotted receiver operating characteristic curves for the diagnosis and prediction of RCC metastasis based on miR-222-3p expression. As shown in Fig. 7C, D, the area under the curve (AUC) value for the diagnosis of RCC using miR-222-3p was 0.9619, while the AUC value for metastasis prediction was 0.6972. This indicated that miR-222-3p is highly valuable for the diagnosis of RCC. Although the diagnostic value of miR-222-3p in RCC metastasis is moderate, its high expression in metastatic RCC suggests a crucial role in promoting metastasis. Finally, we summarized our findings in a schematic diagram (Fig. 7E).

## Discussion

Our study demonstrated that highly invasive RCC cells promote the invasive capability of less invasive RCC cells by secreting miR-222-3p and inducing M2 polarization of macrophages, accelerating the formation of a pre-metastatic niche. Mechanistically, miR-222-3p targets TRPS1, leading to its mRNA degradation and downregulation, which in turn relieves the TRPS1-mediated transcriptional repression of ZEB1. ZEB1 upregulation promotes the migration and invasion of RCC cells by regulating EMT. Additionally, exosomal miR-222-3p can be transported distantly via the bloodstream, promoting M2 polarization of macrophages and creating a pre-metastatic environment favorable for tumor cell colonization. Interestingly, ZEB1 also upregulates miR-222-3p expression at the transcriptional level, forming a positive feedback loop that further promotes the malignant progression of RCC.

Exosomes, which act as mediators of information transmission and material exchange between cells, have demonstrated significance in tumor-related studies [30–32]. They have been used in clinical settings for drug delivery and as diagnostic tools [33]. However, the roles and underlying mechanisms of exosomal miRNAs as diagnostic biomarkers in RCC remain unclear. In this study, we found that miR-222-3p is highly expressed in metastatic RCC and is associated with poor prognosis in these patients. Our findings reveal a previously unknown role of exosomal miR-222-3p in facilitating RCC metastasis and provide insights into the underlying molecular mechanisms.

Emerging evidence suggests that exosomal miRNAs are important regulators of cancer metastasis [34, 35]. In colorectal cancer (CRC), miRNAs may regulate various signaling pathways, including MAPK, Wnt/β-catenin, TGF-β, EMT, and others, thereby influencing metastasis [36]. Moreover, miR-203 has been reported to serve as a prognostic biomarker and predictor of metastasis in human CRC [37]. Similarly, numerous studies have identified miRNAs linked to breast cancer metastasis, functioning as potential prognostic indicators and prediction markers [38]. However, only a few reports have associated miRNAs with RCC. We found that the CM from the highly invasive RCC cell line SN12C-PM6 enhanced the migratory and invasive abilities of less invasive RCC cells. This suggests that the CM from SN12C-PM6 contains components that promote cell invasion. By isolating exosomes from SN12C-PM6 and SN12C cells, followed by miRNA sequencing, we identified miR-222-3p as a pro-metastatic miRNA that was upregulated in highly invasive RCC cells and could be transferred via exosomes to promote cell migration and invasion. Thus, we demonstrated that exosomal miR-222-3p mediates the crosstalk between highly invasive and minimally invasive RCC cells during disease progression. Furthermore, miR-222-3p promotes M2 macrophage polarization, facilitating the formation of a pre-metastatic niche that supports the distant colonization of tumor cells. Evaluating miR-222-3p levels in the circulating exosomes of patients with RCC may reveal its potential as a noninvasive biomarker for the early detection of metastatic progression.

Using miRNA databases, we identified TRPS1 as a potential target of miR-222-3p. TRPS1 is a repressive transcription factor, and together, miR-222-3p and TRPS1 regulate EMT in RCC cells. EMT is a crucial step in cancer cell metastasis, governed by EMT-TFs. We hypothesized that TRPS1 influences the migratory and invasive capabilities of RCC cells by transcriptionally regulating these factors. ZEB1 is a prominent transcriptional regulator involved in EMT and metastasis. Interestingly, we discovered that the ZEB1 promoter region contains binding sites for TRPS1 and that TRPS1 indeed suppresses ZEB1 expression. Additionally, we found that ZEB1 promotes miR-222-3p expression, thereby establishing a positive feedback loop. These findings suggest that the miR-222-3p-TRPS1-ZEB1 axis plays a critical role in the metastasis and malignant progression of RCC.

In summary, this study delineates a novel exosomal miR-222-3p-mediated mechanism that promotes RCC metastasis. Our findings provide a rationale for future investigations into miR-222-3p as a prognostic biomarker and potential therapeutic target to suppress RCC progression. Further studies on the regulatory circuitry governing miR-222-3p expression and secretion may offer additional strategies for managing this deadly disease.

## Materials and methods

### Exosome isolation

In this study, we explored the role of exosomal miR-222-3p in RCC, with a focus on its effects on metastasis and macrophage polarization. SN12C and SN12-PM6 cells were seeded in a 15 cm culture dish. When the cells reached 80–90% confluency, the serum-free medium was replaced, and the cells were cultured for 24 h. The supernatant was then ultracentrifuged at 110,000 × *g* for 70 min to pellet the exosomes. The pellet was washed in phosphate-buffered saline (PBS) and centrifuged again at 110,000 × *g* for 70 min. PBS was removed, and the exosomes re-suspended in 100 µL PBS or nuclease-free water. All centrifugation steps were performed at 4 °C.

### Nanoparticle tracking analysis (NTA)

The size and quantity of exosomes derived from SN12C and SN12-PM6 cells were analyzed using a NanoSight NS300 (Malvern, UK). A 60 s video was captured at a frame rate of 30 frames/second, and the motion of the particles was analyzed using the NTA software.

### Transmission electron microscopy (TEM)

A 20–40 µL solution of exosomes was placed on a copper mesh and post-negatively stained with 1% phosphotungstic acid solution for 10 min. The samples were then dried under incandescent light. Finally, the copper mesh was observed and photographed using a transmission electron microscope.

### Cell culture

Human RCC cell lines SN12C, SN12-PM6, and the human proximal tubular cell line HK2 were obtained from the American Type Culture Collection (ATCC, USA). The mouse renal cancer cell line Renca was purchased from Wuhan Pricella Biotechnology Co., Ltd. Authentication of the cell lines was performed using the Cell Line Authentication IdentiCell STR using STR profiling. Cells were cultured in Dulbecco’s modified Eagle’s medium (DMEM) (SN12C and SN12-PM6), RPMI-1640 (Renca), or MEM medium (HK2), supplemented with 10% fetal bovine serum (FBS; Gibco, USA), 100 U/mL penicillin, and 100 mg/mL streptomycin (Bio Basic Inc., Shanghai, China) in a humidified atmosphere at 37 °C with 5% CO_2_.

### Western blotting analysis

Whole-cell proteins were denatured and subjected to sodium dodecyl sulfate-polyacrylamide gel electrophoresis (SDS-PAGE) on a 10% gradient gel. After electrophoresis, proteins were transferred onto nitrocellulose membranes (Millipore, Billerica, MA, USA). The membranes were blocked with QuickBlock™ Blocking Buffer (Beyotime, Shanghai, China) and incubated overnight at 4 °C with primary antibodies. Secondary antibodies, including horseradish peroxidase-conjugated anti-mouse IgG and anti-rabbit IgG antibodies (Proteintech, Wuhan, China), were used. Immunoreactive protein bands were detected using an ECL Western Blot Detection Kit (Beyotime, Shanghai, China), and proteins were detected using a Bio-Rad ChemiDoc XRS+ System. Bio-Rad Image Lab software was used for densitometric analysis. The primary antibodies used are listed in Table S1.

### Quantitative reverse transcription polymerase chain reaction (qRT-PCR)

Total RNA from tissues and cells was extracted using TRIzol reagent (TaKaRa, Kyoto, Japan). The purified RNA was reverse transcribed using the PrimeScript™ RT reagent kit (Perfect Real Time) (TaKaRa, Kyoto, Japan) to obtain cDNA according to the manufacturer’s protocols. qRT-PCR amplification was then performed using SYBR® Premix Ex Taq™ (Tli RNaseH Plus) (TaKaRa, Kyoto, Japan) on a StepOnePlus™ Real-time PCR system (Thermo Fisher, USA). Quantification was performed using the 2-∆∆Ct method, with GAPDH as the internal control. All primer sequences are listed in Table S2.

### Cell transfection

siRNAs (siNC, siRNA targeting TRPS1, and ZEB1) were obtained from GeneChem (Shanghai, China). Restoration or inhibition of miR-222-3p expression was achieved by transfecting cells with miR-222-3p mimics or mir-222-3p inhibitors, both purchased from GenePharma (Shanghai, China). The sequences of the siRNAs and miRNA mimics are listed in Table S3. Plasmid vectors containing TRPS1, ZEB1, or an empty vector were constructed by GeneChem (Shanghai, China). Cell transfection was carried out using Opti-MEM (Gibco) and Lipofectamine 3000 (Invitrogen) according to the manufacturer’s instructions.

### Wound healing assay

SN12 and SN12-PM6 cells (5 × 10^5^) were digested and plated in six-well plates. After overnight incubation, the cell monolayer was scratched to introduce gaps. The cell culture medium was then replaced with fresh DMEM supplemented with 1% FBS. Images of the wound were captured under a microscope at 0 and 48 h to record the acellular areas at the same location. The results were analyzed using ImageJ software.

### Invasion assays

Cell invasion assays were conducted with a transwell chamber (8 μm pore size; Corning) precoated with or without Matrigel (BD Bioscience). In brief, 1 × 10^5^ cells were suspended in the top chamber with 200 μL of serum-free medium, while 800 μL of culture medium containing 15% FBS was added to the bottom chamber. After 24 h of incubation at 37 °C with 5% CO_2_, the cells that migrated or invaded the underside of the membranes were fixed in 4% polyformaldehyde, stained with 0.5% crystal violet, and five random fields (×40 magnifications) were counted under a microscope.

### Chromatin immunoprecipitation (ChIP)

ChIP was performed using the EZ-ChIP Chromatin Immunoprecipitation Kit (Millipore, USA) following the manufacturer’s protocol. Briefly, formaldehyde was added to cells in 10-cm dishes to a final concentration of 1% and incubated at 37 °C for 15 min to cross-link proteins with DNA. The cells were then sonicated to shear the DNA into fragments of 200–1000 bp. A 20 μL sample was reserved as input for subsequent analysis. The remaining samples were incubated overnight at 4 °C with anti-TRPS1, anti-ZEB1, or negative control antibody (IgG). Protein G agarose beads were added to precipitate the protein-DNA complexes. After washing, the complexes were treated with protease K to digest the proteins, and the supernatant was purified. The primers used for ChIP-qPCR are listed in Table S4.

### Animal experiments

Experimental 4–6-week-old female BALB/c mice were purchased from Weitong Lihua Laboratory Animal Technology Co., Ltd. (Beijing, China) and raised in a specific pathogen-free class environment. Animal experiments in this study were approved and reviewed by the Animal Research Committee of the Academic Medical Center at Huazhong University of Science and Technology. Animal care and handling were performed according to the guidelines of the Institutional Animal Care and Use Committee. For the subcutaneous tumor growth assay, 15 mice were randomly divided into three groups (five mice per group). Renca cells (4 × 10^6^) transfected with the miR-222-3p inhibitor, control, or co-transfected with miR-222-3p mimics were injected into the subcutaneous tissues of the flank. Tumor volumes were measured every 4 days using the formula V = 0.5 × length × width^2^. The endpoint for observation was set when the tumor reached a maximum diameter of 2 cm. After a 28-day monitoring period, all mice were humanely sacrificed under general anesthesia, and the tumors were excised for further analysis. To explore the effects of miR-222-3p on lung metastasis in mice, 4 × 10^6^ Renca cells transfected with the miR-222-3p inhibitor, control, or co-transfected with miR-222-3p mimic were injected into the tail veins of mice. Six weeks later, all mice were sacrificed under general anesthesia after injection. The lungs were collected to count pulmonary metastatic nodules, and the tissues were fixed in formalin and embedded in paraffin for hematoxylin and eosin staining and immunohistochemistry (IHC).

### Microarray analysis of miRNAs

Microarray analysis of miRNAs from RCC tissues and exosomal miRNAs derived from RCC cells was conducted at the Shanghai Biotechnology Corporation (Shanghai, China) using the Agilent Human miRNA 8*60 K V21.0 microarray platform (Agilent Technologies, USA). RCC tissue samples were collected, and exosomes were isolated from the culture media of RCC cell lines. Total RNA, including miRNA, was extracted using the standard miRNA extraction protocol. The extracted RNA samples were labeled and hybridized onto Agilent Human miRNA microarrays according to the manufacturer’s instructions. The arrays were scanned using an Agilent microarray scanner to obtain raw expression data. Quantile normalization was applied to the raw data to ensure consistency and comparability across samples. Normalization and subsequent data processing were performed using the Quantile algorithm implemented in GeneSpring Software 12.6 (Agilent Technologies). The software was also used to filter out low-expression miRNAs and perform initial quality control checks. Normalized data were analyzed to identify differentially expressed miRNAs between RCC tissues and exosomes derived from RCC cells. Hierarchical clustering analysis was performed to visualize the expression patterns of these miRNAs using Pearson’s correlation analysis with Cluster 3.0 software.

### Patients and samples

Human RCC and adjacent normal renal tissues were obtained from 82 patients diagnosed with RCC at the Department of Urology, Union Hospital of Tongji Medical College, Huazhong University of Science and Technology. Among these patients, 52 exhibited distant metastases. The study was approved by the Institutional Review Board of Union Hospital, Tongji Medical College, Huazhong University of Science and Technology. All patients provided consent for the use of their samples and data in experimental studies and scientific publications.

### Immunohistochemistry (IHC)

IHC staining was performed using the streptavidin-biotin-peroxidase complex method. Tissue samples from patients with RCC were subjected to fixation, paraffin embedding, dewaxing, rehydration, and antigen retrieval. The samples were then incubated with antibodies against E-cadherin, N-cadherin, Ki67, TRPS1, ZEB1, and CD206 at 4 °C overnight. This was followed by a 30-min incubation at 37 °C with a secondary biotinylated antibody. Visualization was achieved using a DAB solution, and counterstaining was conducted with hematoxylin. Images were captured using a light microscope.

### Luciferase reporter assay

To identify the binding site between miR-222-3p and TRPS1, cells were transfected with a luciferase construct containing either the wild-type TRPS1 sequence or a mutated version of the binding site. These constructs were co-transfected with either a miR-222-3p mimic or an empty vector. The luciferase vectors were constructed by GenePharma Co. (Shanghai, China). To determine the luciferase activities of ZEB1 and MIR222HG, cells were transfected with luciferase constructs containing a 2000-bp DNA fragment upstream of either the ZEB1 or MIR222HG promoter. Luciferase activity was measured 48 h post-transfection using the Dual-Luciferase Reporter Assay System (Promega, USA), according to the manufacturer’s instructions.

### Statistical analysis

Comparisons between two groups were performed using Student’s *t*-test and *χ*^2^ test. The Kaplan–Meier analysis and log-rank test were used to compare survival rates. Statistical analysis of the microarray data was performed using the *t*-test, analysis of variance, and Fisher’s exact test. Analyses were conducted using SPSS v22.0 and GraphPad Prism 8. All results were from at least three independent experiments and presented as means ± standard deviation (SD). The levels of significance were set at *p < 0.05, **p < 0.01, and ***p < 0.001. NS indicates non-significance.

## Supplementary information

Supplementary Figures

Supplementary methods

Supplementary tables

oringinal Western blots

## Data Availability

The datasets used and/or analyzed during the current study are available from the corresponding author on reasonable request.
