# Supplementary material for: The feedback loop between miR-222-3p and ZEB1 harnesses metastasis in renal cell carcinoma
Source: Cell Death Discov. 2025 Mar 12;11:97. doi: 10.1038/s41420-025-02385-0 (PMC11903659; doi:10.1038/s41420-025-02385-0)
Supplement: Supplementary file 1 — Supplementary Figures [file 41420_2025_2385_MOESM1_ESM.pdf]

The feedback loop between miR-222-3p and ZEB1 harnesses metastasis in renal cell carcinoma

A

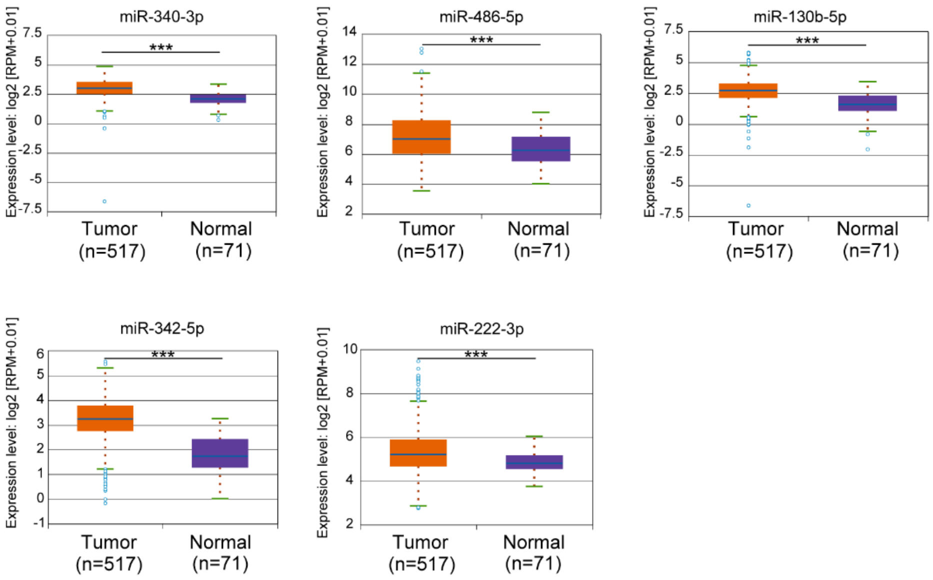

Supplementary Figure 1. Expression of miRNAs in RCC and adjacent normal tissues. A. Expression of miR-340-3p, miR-486-5p, miR-130b-5p, miR-342-5p and miR-222-3p in KIRC and normal tissues obtained through the ENCORI website (https://rnasysu.com/encori/).

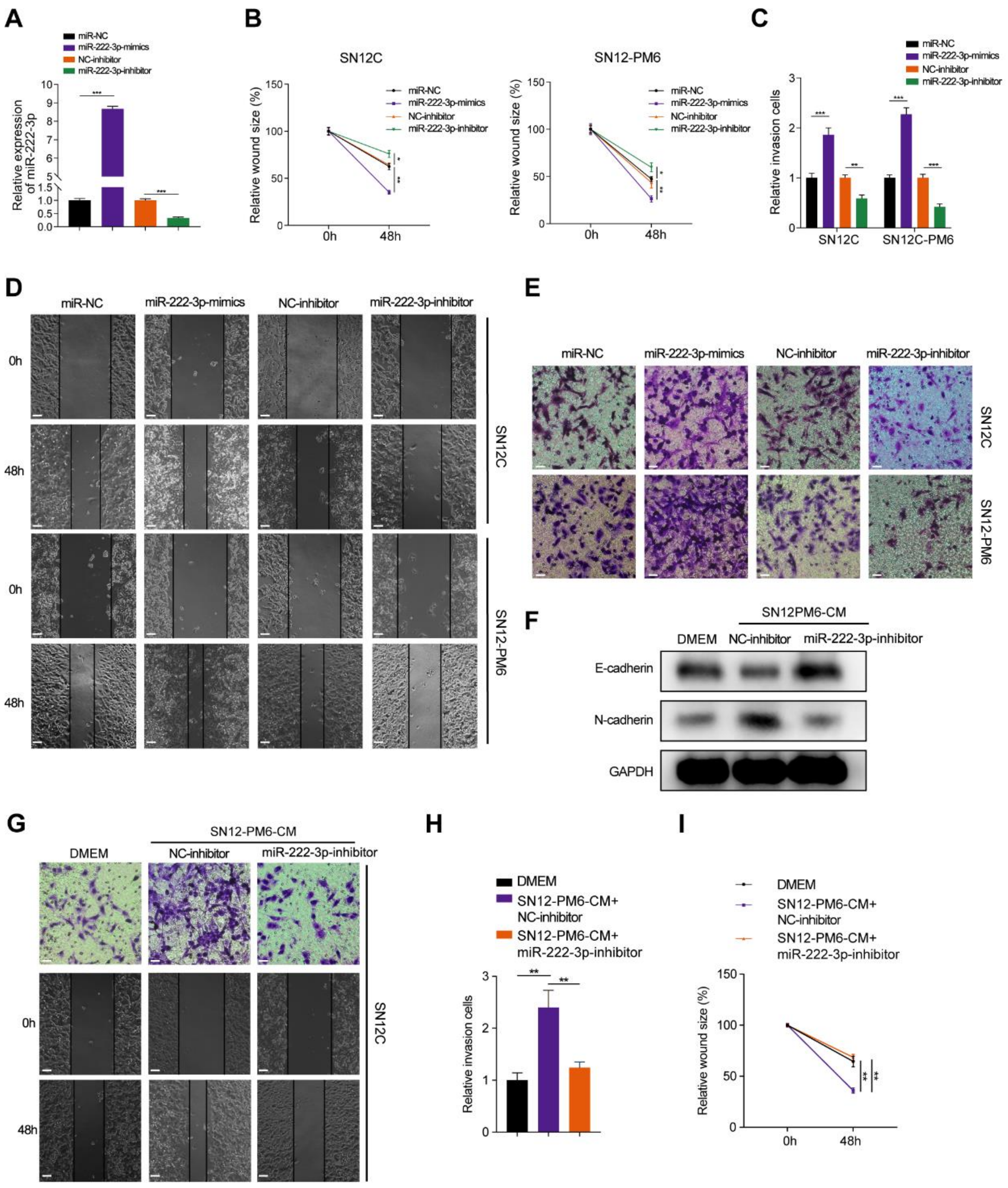

Supplementary Figure 2. miR-222-3p promotes the migration and invasion of RCC cells. A. The overexpression and inhibition efficiency of miR-222-3p in SN12C cells detected by qRT-PCR. Data are presented as the mean ± SD of three independent experiments (n=3). **B.** Quantification of wound healing assays. **C.** Quantification of invasive cells. **D.** Migration of RCC cells after overexpression or inhibition of miR-222-3p. Data are presented as the mean ± SD of three independent experiments (n=3).

Scale bar: 200μm. **E.** Invasion of RCC cells after overexpression or inhibition of miR-222-3p. Data are presented as the mean ± SD of three independent experiments (n=3). Scale bar: 200μm. **F.** Expression levels of E-cadherin and N-cadherin after co-cultivation of CM from SN12-PM6 with SN12C cells pre-transfected with miR-222-3p inhibitor or NC inhibitor. **G.** Migration and invasion of the SN12-PM6 CM co-cultured with SN12C cells pre-transfected with an miR-222-3p or NC inhibitor. Data are presented as the mean ± SD of three independent experiments (n=3). Scale bar: 200μm. **H-I.** Quantification of cell invasion and migration of the figure on the left.

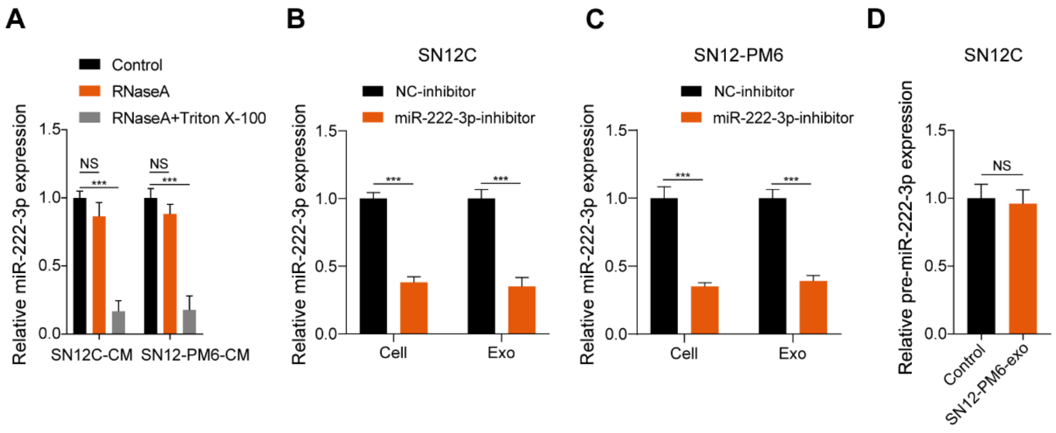

Supplementary Figure 3. mir-222-3p is encapsulated in exosomes. A. Expression of miR-222-3p in CM from SN12C and SN12-PM6 treated with RnaseA alone or in combination with Triton X-100. **B-C.** Expression of miR-222-3p in SN12C (B) or SN12-PM6 (C) cells and their derived exosomes after transfection with miR-222-3p inhibitor or NC-inhibitor. **D.** The effect on the expression of pre-miR-222-3p after co-cultivation of SN12-PM6-exo with SN12C cells. Data are presented as the mean ± SD of three independent experiments (n=3).

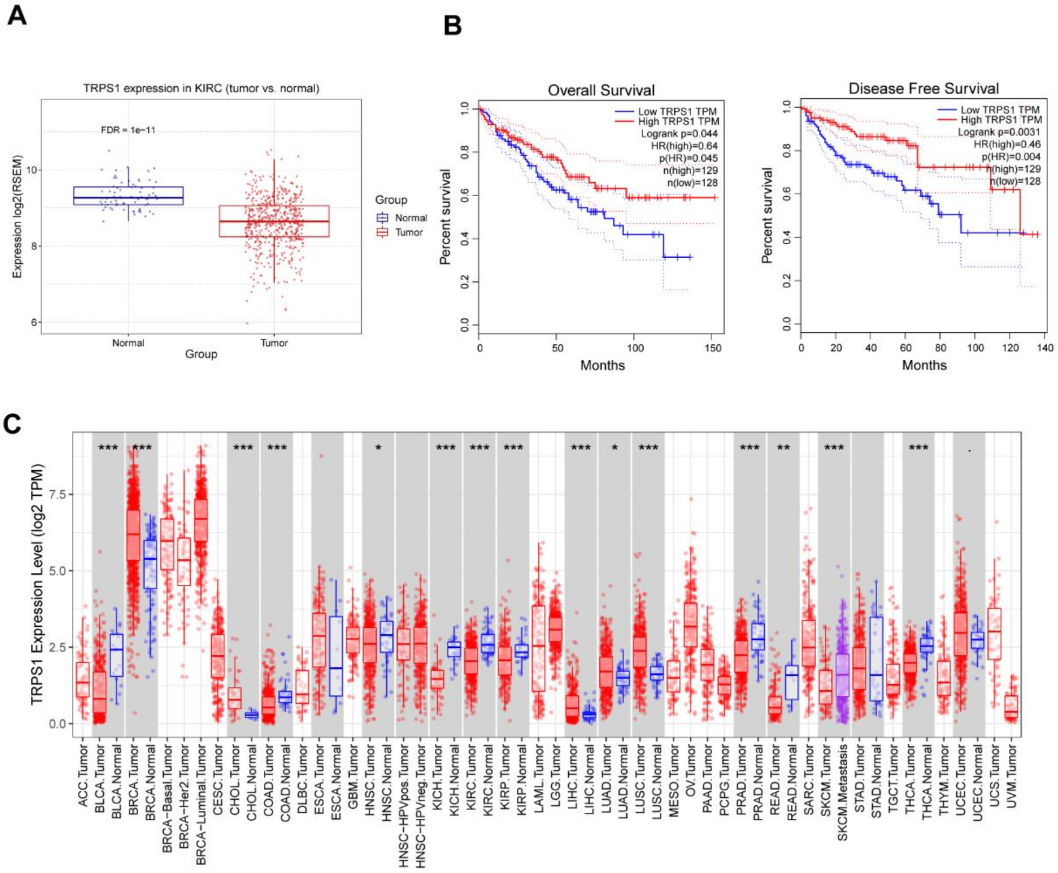

Supplementary Figure 4. Low TRPS1 expression in RCC is associated with poor survival prognosis in patients. A. Expression of TRPS1 in KIRC tumors and normal tissues. **B.** Survival curves of total survival and disease-free survival obtained from the GEPIA database (http://gepia.cancer-pku.cn/) for TRPS1 expression. **C.** Pan-cancer expression bar chart of TRPS1 obtained from the TIMER 2.0 database (http://timer.cistrome.org/).

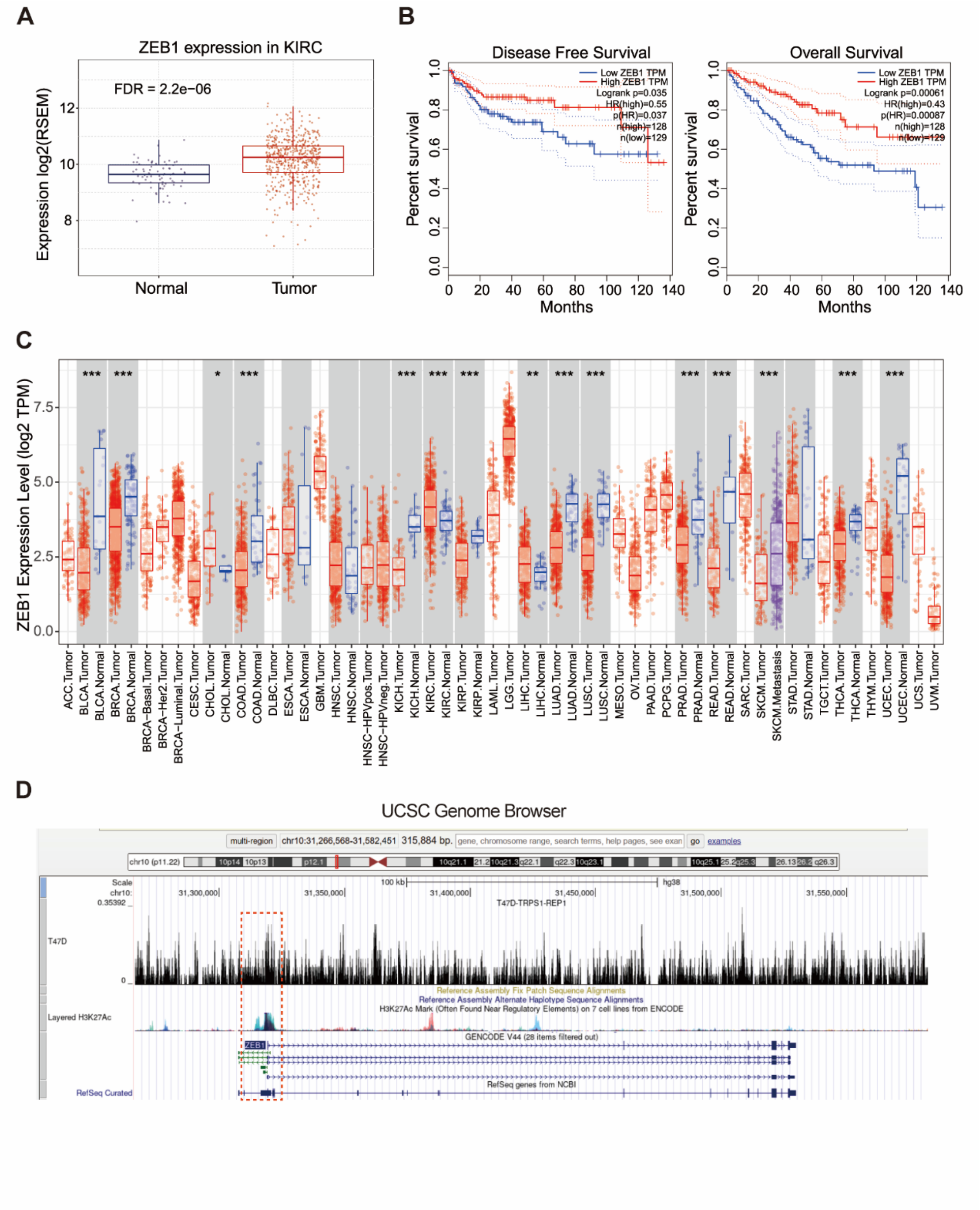

Supplementary Figure 5. High ZEB1 expression in RCC is associated with poor survival prognosis in patients. A. Expression of ZEB1 in KIRC tumors and normal tissues. **B.** Survival curves of total survival and disease-free survival obtained from the GEPIA database for ZEB1 expression. **C.** Pan-cancer expression bar chart of

ZEB1 obtained from the TIMER 2.0 database. **D.** Binding peak map of TRPS1 in the ZEB1 promoter region analyzed by UCSC Genome Browser.

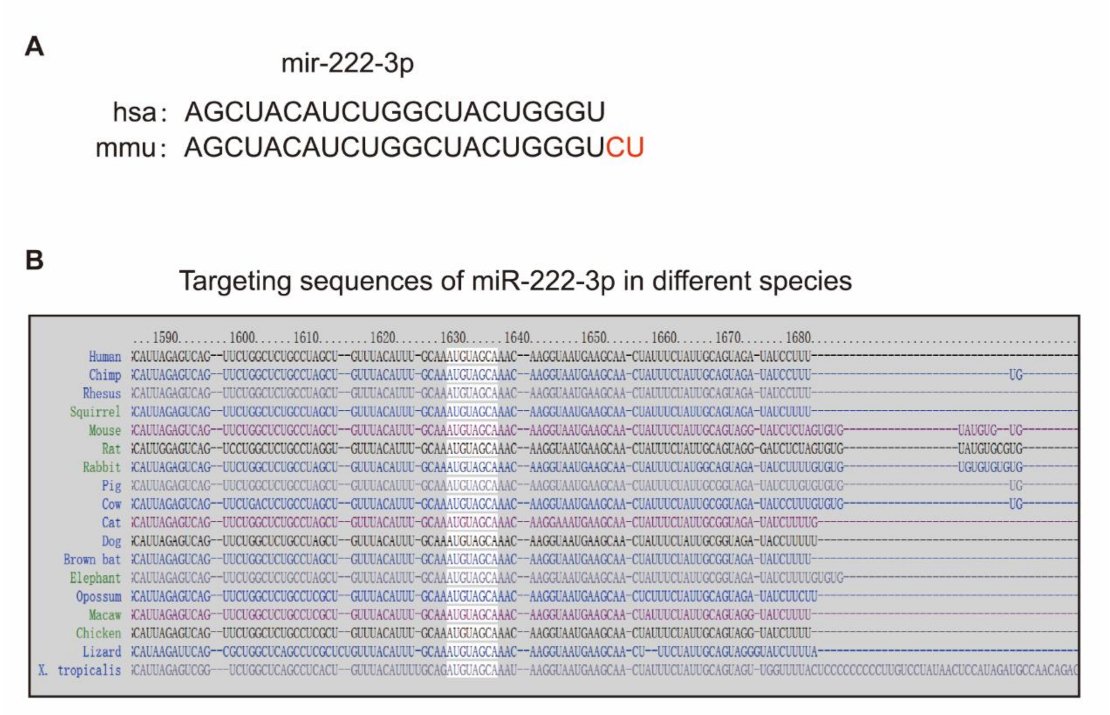

Supplementary Figure 6. miR-222-3p is conserved across species. A. Sequence of miR-222-3p in human and mouse obtained from the miRBase database. **B.** Binding sequences of miR-222-3p in different species analyzed by the targetscan database.
