# Supplementary material for: The feedback loop between miR-222-3p and ZEB1 harnesses metastasis in renal cell carcinoma
Source: Cell Death Discov. 2025 Mar 12;11:97. doi: 10.1038/s41420-025-02385-0 (PMC11903659; doi:10.1038/s41420-025-02385-0)
Supplement: Supplementary file 2 — Supplementary methods [file 41420_2025_2385_MOESM2_ESM.docx]

**Exosome isolation**

SN12C and SN12-PM6 cells were planted on 15cm culture dish. When the confluence of the cells reached 80%-90%, replaced the serum-free medium and continued to culture for 24 hours. The supernatant was ultra-centrifuged at 110,000 × g for 70 min to pellet the exosomes. The pellet was washed in phosphate-buffered saline (PBS) centrifuged again at 110,000 × g for 70 min. The PBS was removed and the exosomes re-suspended in 100 µl PBS or nuclease-free water. All centrifugation steps were performed at 4°C.

**Nanoparticle tracking analysis (NTA)**

The size and quantitation of SN12C and SN12-PM6 cells-derived exosomes were analyzed using a NanoSight NS300 (Malvern, UK). A video with a duration of 60 seconds was taken at a frame rate of 30 frames/second, and the motion of particles was analyzed by using NTA software.

**Transmission electron microscopy (TEM)**

A 20-40 µl solution of exosomes were placed on a copper mesh and post-negatively stained with 1% phosphotungstic acid solution for 10 min. The sample was then dried under incandescent light. Finally, the copper mesh was observed and photographed under a transmission electron microscope.

**Cell culture**

Human HCC cell lines SN12C, SN12-PM6 and human proximal tubular cell line HK2 were obtained from American Type Culture Collection (ATCC, USA). The mouse renal cancer cell line Renca was purchased from Wuhan Pricella Biotechnology Co., Ltd. Authentication of cell lines was performed by Cell Line Authentication IdentiCell STR using STR profiling. Cells were cultured in Dulbecco’s modified Eagle’s medium (DMEM) (SN12C and SN12-PM6), RPMI-1640 (Renca) or MEM medium (HK2), supplemented with 10% FBS (Gibco, USA), 100 U/mL penicillin and 100 mg/ml streptomycin (Bio Basic Inc., Shanghai, China) in a humidified atmosphere at 37℃, 5% CO2.

**Western Blotting Analysis**

Whole cells proteins were denatured and subjected to SDS-PAGE (on a 10% gradient gel) analysis. After electrophoresis, the proteins were electrotransferred onto a nitrocellulose membrane (Millipore, USA). After blocking with QuickBlock™ Blocking Buffer (Beyotime, Shanghai, China), the primary antibodies were incubated the membrane with overnight at 4°C. HRP-conjugated anti-mouse IgG and anti-rabbit IgG antibodies (Proteintech, Wuhan, China) were used as the secondary antibodies. The immunoreactive protein bands were detected by ECL Western Blot Detection Kit (Beyotime, Shanghai, China) and Proteins were detected using a Bio-Rad ChemiDoc XRS+ System. Bio-Rad Image Lab software was used for densitometric analysis. The primary antibodies were listed in Supplementary Table 1.

**Quantitative real-time PCR (qRT-PCR)**

Total tissues and cells RNA were extracted using TRIzol reagent (TaKaRa, Kyoto, Japan). The purified RNA was reverse transcribed with PrimeScript™ RT reagent Kit (Perfect Real Time) (TaKaRa, Kyoto, Japan) to obtain cDNA according to the manufacturers’ protocols. Next, real-time polymerase chain reaction (PCR) amplification was performed with SYBR® Premix Ex Taq™ (Tli RNaseH Plus) (TaKaRa, Kyoto, Japan) on a StepOnePlus™ Real-time PCR system (Thermo Fisher, USA). Quantification was performed according to the 2-∆∆Ct method. GAPDH was used as an internal control. All primer sequences are listed in Supplementary Table 2.

**Cell transfection**

siRNAs (siNC, siRNA targeting TRPS1, ZEB1) were conducted by GeneChem (Shanghai, China). Restoration or inhibition of miR-222-3p expression was achieved by transfecting cells with miR-222-3p mimics or mir-222-3p inhibitor, which were purchased from GenePharma (Shanghai, China). The sequences of siRNAs and miRNA mimic referred above were listed in Supplementary Table 3. The plasmid vector containing TRPS1, ZEB1 and empty vector were conducted by GeneChem (Shanghai, China). Opti-MEM (Gibco) and Lipofectamine 3000 (Invitrogen) were used for cell transfection according to the manufacturer's instructions.

**Wound healing assay**

5 x 10^5^ SN12 and SN12-PM6 cells were digested and planted on a 6-well plate. After overnight incubation, the cell monolayer was scratched to introduce a gap. Cell medium was replaced with fresh DMEM containing 1% FBS. We took pictures under the microscope at 0 and 48 hours to record the acellular areas at the same location. Image J software is used to analyze the results.

**Invasion assays**

Cell invasion assays were performed with a transwell chamber (8 μm pore size; Corning) precoated with or without Matrigel (BD Bioscience), respectively. In brief, 1×10^5^ cells were suspended in the top chamber with 200 μL of serum-free medium, whereas 800 μL of the culture medium containing 15% FBS were added into the bottom chamber. After incubating for 24 h in 37℃, 5% CO2, the cells which migrated or invaded the underside of the membranes were fixed in 4% polyformaldehyde, stained with 0.5% crystal violet, and five random fields (×40 magnifications) were counted under microscope.

**Chromatin immunoprecipitation (ChIP)**

ChIP was performed using the EZ-ChIP™ Chromatin Immunoprecipitation Kit (Millipore, USA) following the manufacturer's protocol. Briefly, formaldehyde was added to 10 cm dishes of cells to a final concentration of 1% and incubated at 37°C for 15 minutes to cross-link proteins with DNA. Cells were then sonicated to shear the DNA into fragments of 200-1000 bp. A 20 μl sample was reserved as input for later analysis. The remaining samples were incubated overnight at 4°C with anti-TRPS1, anti-ZEB1 antibodies, or a negative control antibody (IgG). Protein G agarose beads were added to precipitate the protein-DNA complexes. After washing, the complexes were treated with protease K to digest the proteins, and the supernatant was purified. Primers for ChIP-qPCR are listed in in Supplementary Table 4.

**Animal experiments**

Experimental 4-6 weeks old female BALB/c mice were purchased from Weitong Lihua (Beijing, China) Laboratory Animal Technology Co., Ltd. All mice were raised in a SPF-class environments. The animal experiments in this study were approved and reviewed by the Animal Research Committee of the Academic Medical Center at Huazhong University of Science and Technology. Care and handling of the animals were following the guidelines of Institutional and Animal Care and Use Committees. For the subcutaneous tumor growth assay, fifteen mice were randomly divided into three groups (5 mice per group). 4 × 10^6^ Renca cells transfected with miR-222-3p inhibitor, control or co-transfected with miR-222-3p mimics were injected into the subcutaneous tissues of of the flank. Tumor volumes were assessed every four days using the formula V = 0.5 × length × width^2. The endpoint for observation was set when the tumors reached a maximum diameter of 2 cm. After a 28-day monitoring period, all mice were humanely sacrificed under general anesthesia, and the tumors were excised for further analysis. To explore the effects of miR-222-3p on lung metastasis in mice, 4 × 10^6^ Renca cells transfected with miR-222-3p inhibitor, control or co-transfected with miR-222-3p mimics were injected into the tail vein of mice. Six weeks later, all mice were sacrificed under general anesthesia after injection. The lungs of mice were collected for counting the pulmonary metastatic nodules. The lungs fixed in formalin were processed with paraffin embedding for Hematoxylin and Eosin (H&E) staining and IHC.

**Microarray analysis of miRNAs**

Microarray analysis of miRNAs from RCC tissues and exosomal miRNAs derived from RCC cells was conducted at Shanghai Biotechnology Corporation (Shanghai, China) using the Agilent Human miRNA 8*60K V21.0 microarray platform (Agilent Technologies, USA). RCC tissue samples were collected, and exosomes were isolated from the culture media of RCC cell lines. Total RNA, including miRNAs, was extracted using a standard miRNA extraction protocol. Extracted RNA samples were labeled and hybridized onto the Agilent Human miRNA microarrays according to the manufacturer's instructions. The arrays were then scanned using an Agilent microarray scanner to obtain raw expression data. Quantile normalization was applied to the raw data to ensure consistency and comparability across samples. This normalization and subsequent data processing were performed using the Quantile algorithm implemented in GeneSpring Software 12.6 (Agilent Technologies). The software was also used to filter out low-expressing miRNAs and to perform initial quality control checks. The normalized data were analyzed to identify differentially expressed miRNAs between the RCC tissues and the RCC cell-derived exosomes. Hierarchical clustering analysis was performed to visualize the expression patterns of these miRNAs, using Pearson's correlation analysis with Cluster 3.0 software.

**Patients and samples**

Human RCC tissues and adjacent normal renal tissues were obtained from 82 patients diagnosed with RCC at the Department of Urology, Union Hospital of Tongji Medical College, Huazhong University of Science and Technology. Among these cases, 52 exhibited distant metastasis. This study was approved by the Institutional Review Board at Union Hospital, Tongji Medical College, Huazhong University of Science and Technology. All patients provided consent for the use of their sample data in experimental studies and scientific publications.

**Immunohistochemistry (IHC)**

IHC staining was performed utilizing the streptavidin-biotin-peroxidase complex methodology. In brief, tissue samples from pancreatic cancer were subjected to fixation, paraffin embedding, dewaxing, rehydration, and antigen retrieval processes. Subsequently, these samples were incubated with antibodies against E-cadherin, N-cadherin, Ki67, TRPS1, ZEB1, or CD206 at 4°C overnight. This was followed by a 30-minute incubation at 37°C with a secondary biotinylated antibody. Visualization was achieved using a DAB solution, and counterstaining was conducted with hematoxylin. Images were captured utilizing a light microscope.

**Luciferase reporter assay**

To identify the binding site between miR-222-3p and TRPS1, cells were transfected with a luciferase construct containing either the wild-type TRPS1 sequence or a mutated version of the binding site. These constructs were cotransfected with either a miR-222-3p mimic or an empty vector. The luciferase vectors were constructed by GenePharma Co. (Shanghai, China). To determine the luciferase activities of ZEB1 or MIR222HG, cells were transfected with luciferase constructs containing a 2000-bp DNA fragment upstream of either the ZEB1 or MIR222HG promoter. Luciferase activities were measured using the Dual-Luciferase Reporter Assay System (Promega, USA) according to the manufacturer's instructions 48 hours post-transfection.

**Statistical analysis**

Comparisons between the two groups were performed using the student t test, χ2 test. Kaplan-Meier analysis and a log-rank test were used to compare the different survival rates. Statistical analysis of Microarray data was summarized using the t test, ANOVA and Fisher’s exact test. SPSS v22.0 and GraphPad Prism 8 were used for analyses, and all results were at least three independent experiments and were presented as means ± standard deviation (SD). The level of significance was set at * p<0.05, ** p<0.01, *** p<0.001. N.S. indicates non‐significance.
