# Supplementary material for: The feedback loop between miR-222-3p and ZEB1 harnesses metastasis in renal cell carcinoma
Source: Cell Death Discov. 2025 Mar 12;11:97. doi: 10.1038/s41420-025-02385-0 (PMC11903659; doi:10.1038/s41420-025-02385-0)
Supplement: Supplementary file 3 — Supplementary tables [file 41420_2025_2385_MOESM3_ESM.docx]

**Supplementary Table 1.** The antibodies used in this article.

| ANTIBODIES | SOURCE | IDENTIFIER |
| --- | --- | --- |
| E-cadherin | Proteintech | 20874-1-AP |
| N-cadherin | Proteintech | 20874-1-AP |
| CD63 | Proteintech | 25682-1-AP |
| CD81 | Proteintech | 27855-1-AP |
| TRPS1 | Proteintech | 21938-1-AP |
| ZEB1 | Proteintech | 21544-1-AP |
| GAPDH | Proteintech | 10494-1-AP |

**Supplementary Table 2.** The sequences of PCR primers.

| **Primer** | **Sequence** |
| --- | --- |
| miR-340-3p | Forward: 5'- TCCGTCTCAGTTATTTATAGC -3' |
|  | Reverse: 5'- GCGAGCACAGAATTAATACGAC -3' |
| miR-342-5p | Forward: 5'- AGGGGTGCTATCTGTGATTGA -3' |
|  | Reverse: 5'- GCGAGCACAGAATTAATACGAC -3' |
| miR-486-5p | Forward: 5'- TCCGTGTACTGAGCTGCCCCGAG -3' |
|  | Reverse: 5'- GCGAGCACAGAATTAATACGAC -3' |
| miR-130b-5p | Forward: 5'- ACTCTTTCCCTGTTGCACTAC -3' |
|  | Reverse: 5'- GCGAGCACAGAATTAATACGAC -3' |
| miR-222-3p | Forward: 5'- AGCTACATCTGGTCACTGGGT -3' |
|  | Reverse: 5'- GCGAGCACAGAATTAATACGAC -3' |
| CDKN1B | Forward: 5'-ATCACAAACCCCTAGAGGGCA |
|  | Reverse: 5'- GGGTCTGTAGTAGAACTCGGG |
| GNAI3 | Forward: 5'-GACGGCTAAAGATTGACTTTGGG |
|  | Reverse: 5'- GACGGCTAAAGATTGACTTTGGG |
| MYLIP | Forward: 5'-GCAGGCGACTGGGAATCATAG |
|  | Reverse: 5'- GCAGGCGACTGGGAATCATAG |
| TMCC1 | Forward: 5'-AAGTCTGGTCAGGAGATGACAG |
|  | Reverse: 5'- AAGTCTGGTCAGGAGATGACAG |
| SUN2 | Forward: 5'-TGACGTGCCTGACGTATGG |
|  | Reverse: 5'- AAATGTGGCGATGAGTCTCTG |
| ESR1 | Forward: 5'-CCCACTCAACAGCGTGTCTC |
|  | Reverse: 5'- CGTCGATTATCTGAATTTGGCCT |
| PAIP2 | Forward: 5'-TCTCCCACAAACTATGGACCA |
|  | Reverse: 5'- TGCATTTGGATTCAGATTGCTCT |
| YWHAG | Forward: 5'-AGCCACTGTCGAATGAGGAAC |
|  | Reverse: 5'- CTGCTCAATGCTACTGATGACC |
| TRPS1 | Forward: 5'-AGCCCCAGTAAGGGAGGAAA |
|  | Reverse: 5'- GGGTGCAGGCCATATCTTGAG |
| PDCD10 | Forward: 5'-GCCCCTCTATGCAGTCATGTA  Reverse: 5'- AGCCTTGATGAAAGCGGCTC |
| ETS1 | Forward: 5'-GATAGTTGTGATCGCCTCACC |
|  | Reverse: 5’-GTCCTCTGAGTCGAAGCTGTC |
| FOS | Forward: 5'-CCGGGGATAGCCTCTCTTACT |
|  | Reverse: 5’-CCAGGTCCGTGCAGAAGTC |
| ZEB1 | Forward: 5'-GATGATGAATGCGAGTCAGATGC |
|  | Reverse: 5’-ACAGCAGTGTCTTGTTGTTGT |
| ZEB2 | Forward: 5'-CAAGAGGCGCAAACAAGCC  Reverse: 5’-GGTTGGCAATACCGTCATCC |
| SNAIL1 | Forward: 5'-TCGGAAGCCTAACTACAGCGA |
|  | Reverse: 5’-AGATGAGCATTGGCAGCGAG |
| SNAIL2 | Forward: 5'-CGAGTGGTTCTTCTGCGCTA |
|  | Reverse: 5’-GGGCTGCTGGAAGGTAAACT |
| TWIST1 | Forward: 5'-GTCCGCAGTCTTACGAGGAG |
|  | Reverse: 5’-GCTTGAGGGTCTGAATCTTGCT |
| TWIST2 | Forward: 5'-GCGCAAGTGGAATTGGGATG |
|  | Reverse: 5’-CGGGTCTTCTGTCCGATGTC |
| FOXC2 | Forward: 5'-CCTCCTGGTATCTCAACCACA |
|  | Reverse: 5’-GAGGGTCGAGTTCTCAATCCC |
| SOX4 | Forward: 5'-AGCGACAAGATCCCTTTCATTC |
|  | Reverse: 5’-CGTTGCCGGACTTCACCTT |
| SOX9 | Forward: 5'-AGCGAACGCACATCAAGAC |
|  | Reverse: 5’-CTGTAGGCGATCTGTTGGGG |
| CD206 | Forward: 5’-GGGTTGCTATCACTCTCTATGC-3’ |
|  | Reverse: 5’-TTTCTTGTCTGTTGCCGTAGTT-3’ |
| CD163 | Forward: 5’-TTTGTCAACTTGAGTCCCTTCAC-3’ |
|  | Reverse: 5’-TCCCGCTACACTTGTTTTCAC-3’ |
| ARG1 | Forward: 5’-GTGGAAACTTGCATGGACAAC-3’ |
|  | Reverse: 5’-AATCCTGGCACATCGGGAATC-3’ |
| IL-10 | Forward: 5’-GACTTTAAGGGTTACCTGGGTTG-3’ |
|  | Reverse: 5’-TCACATGCGCCTTGATGTCTG-3’ |
| TGF-β | Forward: 5'-CAATTCCTGGCGATACCTCAG |
|  | Reverse: 5’-GCACAACTCCGGTGACATCAA |
| IL-6 | Forward: 5’-AATAACCACCCCTGACCCAAC-3’ |
|  | Reverse: 5’-ACATTTGCCGAAGAGCCCT-3’ |
| CCL16 | Forward: 5'-ACAGAAAGGCCCTCAACTGTC |
|  | Reverse: 5’-TCCTTGATGTACTCTTGGACCC |
| CCL18 | Forward: 5'-AACAAAGAGCTCTGCTGCCT |
|  | Reverse: 5’-CTGGGGGCTGGTTTCAGAAT |
| IL-1β | Forward: 5'-ATGATGGCTTATTACAGTGGCAA |
|  | Reverse: 5’-GTCGGAGATTCGTAGCTGGA |
| U6 snRNA | Forward: 5'- CTCGCTTCGGCAGCACA -3' |
|  | Reverse: 5'- AACGCTTCACGAATTTGCGT -3' |
| GAPDH | Forward: 5’-TGAACGGGAAGCTCACTGG-3’ |
|  | Reverse: 5’-TCCACCACCCTGTTGCTGTA-3’ |

**Supplementary Table 3.** The sequence of siRNA and miR-222-3p mimic/inhibitor

| siRNA | Sequence (5′-3′) |
| --- | --- |
| TRPS1-siRNA#1 sense | UUAUCAGAAAUGUUAUCGCUU |
|  | GCGAUAACAUUUCUGAUAAAG |
| TRPS1-siRNA#2 sense | UUUUUACAAUUAUUAAUUCUA |
|  | GAAUUAAUAAUUGUAAAAAGC |
| ZEB1-siRNA#1 sense | AAUUUGUAACGUUAUUGCGCC |
|  | CGCAAUAACGUUACAAAUUAU |
| ZEB1-siRNA#2 sense | UGUAACACUUUCUUCUUCCAC |
|  | GGAAGAAGAAAGUGUUACAGA |
| miR-222-3p mimic | AGCUACAUCUGGCUACUGGGU |
|  | ACCCAGUAGCCAGAUGUAGCU |
| miR-222-3p inhibitor | ACCCAGUAGCCAGAUGUAGCU |

**Supplementary Table 4.** Primers used for ChIP

|  | **Sequence** |
| --- | --- |
| CHIP: ZEB1 | S1: F 5'-AAACTGGCTCACAGCTCCTT-3' |
|  | R 5'-GTCTTGAACTCCTGACCTTAG-3' |
|  | S2: F 5'-CTTTAACACAAGTCAACTCTCAGG-3' |
|  | R 5'-GCAAGACAGAAAAGTCATTTCC-3' |
| CHIP: MIR222HG | F: 5'-TAATACGACTCACTATAGGGATTTTATTATTGTT-3' |
|  | R: 5'-TCGGTCTCACCCCTCAGTTTCT-3' |
