# Supplementary figures and images for: The feedback loop between miR-222-3p and ZEB1 harnesses metastasis in renal cell carcinoma
Source: Cell Death Discov. 2025 Mar 12;11:97. doi: 10.1038/s41420-025-02385-0 (PMC11903659; doi:10.1038/s41420-025-02385-0)

Figure 2

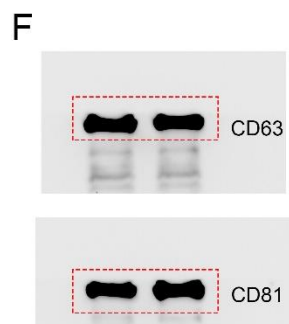

Figure 3

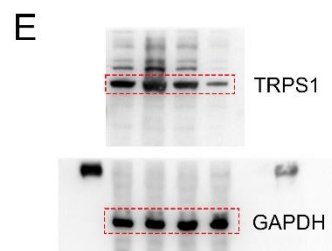

Figure 4

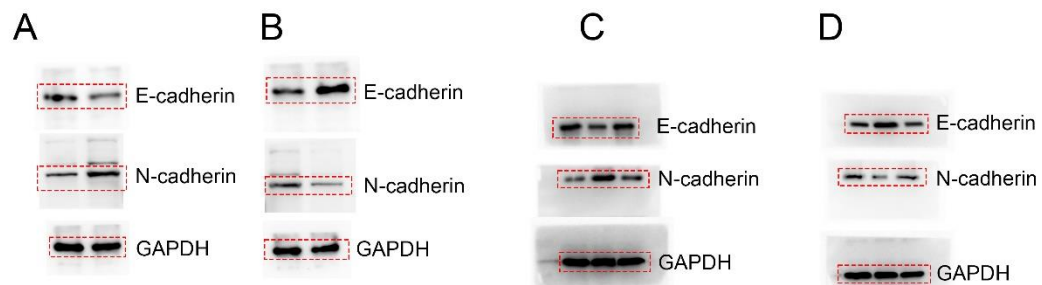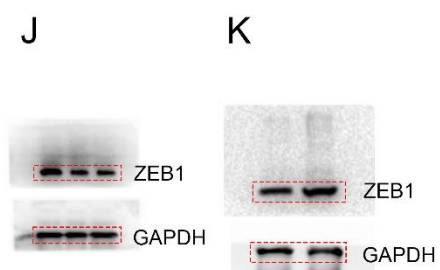

Supplementary Fig 2

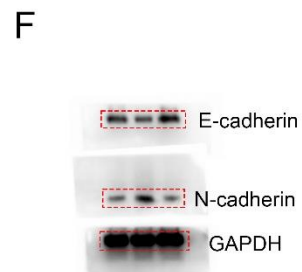

Supplement: Supplementary file 4 — oringinal Western blots [file 41420_2025_2385_MOESM4_ESM.pdf]
